# Immunodominant Linear B-Cell Epitopes of SARS-CoV-2 Spike, Identified by Sera from K18-hACE2 Mice Infected with the WT or Variant Viruses

**DOI:** 10.3390/vaccines10020251

**Published:** 2022-02-07

**Authors:** Yinon Levy, Ron Alcalay, Anat Zvi, Efi Makdasi, Eldar Peretz, Tal Noy-Porat, Theodor Chitlaru, Michal Mandelboim, Ohad Mazor, Ronit Rosenfeld

**Affiliations:** 1 Department of Biochemistry and Molecular Genetics, Israel Institute for Biological Research, Ness-Ziona 7410001, Israel; rona@iibr.gov.il (R.A.); anatz@iibr.gov.il (A.Z.); efim@iibr.gov.il (E.M.); eldarp@iibr.gov.il (E.P.); taln@iibr.gov.il (T.N.-P.); theodorc@iibr.gov.il (T.C.); 2 The Central Virology Laboratory, Israel Ministry of Health, Tel Hashomer, Ramat Gan 5266202, Israel; Michal.Mandelboim@sheba.health.gov.il; 3 Department of Epidemiology and Preventive Medicine, School of Public Health, Sackler Faculty of Medicine, Tel-Aviv University, Tel-Aviv 6997801, Israel

**Keywords:** COVID-19, SARS-CoV-2, epitope mapping, linear epitopes, K18-hACE2

## Abstract

SARS-CoV-2 surface spike protein mediates the viral entry into the host cell and represents the primary immunological target of COVID-19 vaccines as well as post-exposure immunotherapy. Establishment of the highly immunogenic B-cell epitope profile of SARS-CoV-2 proteins in general, and that of the spike protein in particular, may contribute to the development of sensitive diagnostic tools and identification of vaccine` candidate targets. In the current study, the anti-viral antibody response in transgenic K18-hACE-2 mice was examined by implementing an immunodominant epitope mapping approach of the SARS-CoV-2 spike. Serum samples for probing an epitope array covering the entire spike protein were collected from mice following infection with the original SARS-CoV-2 strain as well as the B.1.1.7 Alpha and B.1.351 Beta genetic variants of concern. The analysis resulted in distinction of six linear epitopes common to the humoral response against all virus variants inspected at a frequency of more than 20% of the serum samples. Finally, the universality of the response was probed by cross-protective in vitro experiments using plaque-reducing neutralization tests. The data presented here has important implications for prediction of the efficacy of immune countermeasures against emerging SARS-CoV-2 variants.

## 1. Introduction

Since the onset of the current coronavirus pandemic, limiting the dissemination of SARS-CoV-2, the etiological agent of the COVID-19, represents an objective of utmost public health priority. The recent licensing of several vaccines enabled, to some extent, control of the pandemic in a limited number of developed countries. However, global expansion of the virus amongst unvaccinated individuals and the emergence of variants have raised concerns with respect to the efficacy of these COVID-19 vaccines [1,2]. SARS-CoV-2 variants that have widely propagated, associated with their enhanced transmissibility, morbidity and/or reduced susceptibility to vaccination or immunotherapies, have been classified by the World Health Organization (WHO) as variants of concern (VOCs, coined chronologically by the Greek alphabet) (https://www.who.int/en/activities/tracking-SARS-CoV-2-variants/, as for 1 January 2022).

Although SARS-CoV-2 VOCs accumulated mutations throughout their genomes, the mutations that map to the spike glycoprotein attract most of the scientific attention, due to the central role that the spike has in the viral pathogenesis and considering that the spike represents the antigen targeted by all FDA-approved vaccines against COVID-19. The spike receptor binding domain (RBD) within the subunit 1 (S1) mediates the binding of the virus to the host angiotensin converting enzyme 2 (ACE2) [3,4]. The subsequent cleavage and separation of the S1 and S2 subunits and the formation of the S2 fusion complex enables the viral entry into affected cells [5]. As a consequence of the central role of RBD in the internalization of the virus into host cells, the titers of RBD-specific antibodies in human sera (from infected or convalescent individuals) significantly correlate with the neutralizing potential of the sera [6,7]. Apart from the RBD, additional domains of the S1 subunit, in particular the N-terminal domain (NTD), can also elicit generation of antibodies [8,9], some of which have been documented to also exhibit virus-neutralizing ability, suggesting that they also participate in the recognition and engagement of the cellular receptor [10,11,12,13]. Accumulating structural, biochemical and immunological data revealed that the NTD and to a lesser extent the RBD of the VOCs underwent significant conformational changes that lowered the efficacy of antibodies directed against these regions of the spike glycoprotein [14,15]. These observations may affect the efficacy of the FDA-approved vaccines, as all of them were generated against the Wuhan Hu-1 initial isolate spike glycoprotein [16]. 

The identification of immunodominant B-cell epitopes in the SARS-CoV-2 spike glycoprotein represents an important information for dissecting the host protective immunity against the virus. The immune-dominant epitope landscape of the spike may be correlated to the efficacy of the immune response and/or to the severity of the illness. Thus, it may contribute to the development of efficient tests for evaluation of the potential protective characteristic of immune response elicited by a given vaccine or exhibited by infected/recovering individuals. Optimization of the epitope architecture of the spike may be beneficial for the improvement of vaccine efficacy against the VOCs. The human antibody response against the SARS-CoV-2 glycoprotein was extensively investigated using several approaches such as bioinformatics-based prediction [17,18,19,20,21] as well as peptide-based microarray experiments [9,11,22,23,24,25]. Nevertheless, most of these studies were done by sera obtained from individuals infected with the Wuhan Hu-1 isolate prior to the emergence of the VOCs.

In the current study, we implemented the K18-hACE2 transgenic murine model for directly inspecting the epitope landscape characterizing the antibody response against the wild-type (WT) SARS-CoV-2 spike protein as well as its mutated versions, represented by the B.1.1.7 Alpha and the B.1.351 Beta variants of concern (VOCs). 

## 2. Materials and Methods

### 2.1. Virus Strains

Wild-type (WT) SARS-CoV-2 strain BavPat1/2020 was kindly provided by Prof. Dr. Christian Drosten (Charité, Berlin, Germany) through the European Virus Archive—Global (EVAg Ref-SKU: 026V-03883).

SARS-CoV-2 B.1.1.7 (501Y.V1; Alpha) and SARS-CoV-2 B.1.351 (501Y.V2; Beta) variants were isolated, sequence-identified and cultured as recently described [14]. 

Handling and working with SARS-CoV-2 was conducted in BSL3 facility in accordance with the biosafety guidelines of the IIBR.

### 2.2. Serum Samples

#### 2.2.1. Humans

Anonymized surplus blood samples of COVID-19 patients with persistent critical illness >21 days after first day of symptom onset and taken as part of routine care (St Thomas’ Hospital, London, UK) were retrieved at the point of being discarded (April 2020). Work was undertaken in accordance with UK Research Ethics Committee (REC) approval for laboratory research projects investigating the immune response to COVID-19 infection with academic collaborators (REC reference 20/SC/0310). Samples were treated in accordance with the biosafety guidelines of the IIBR in BL3 facility. Sera samples were heat-inactivated (20 min at 60 °C) prior to use.

#### 2.2.2. Mice

**Figure 1:**
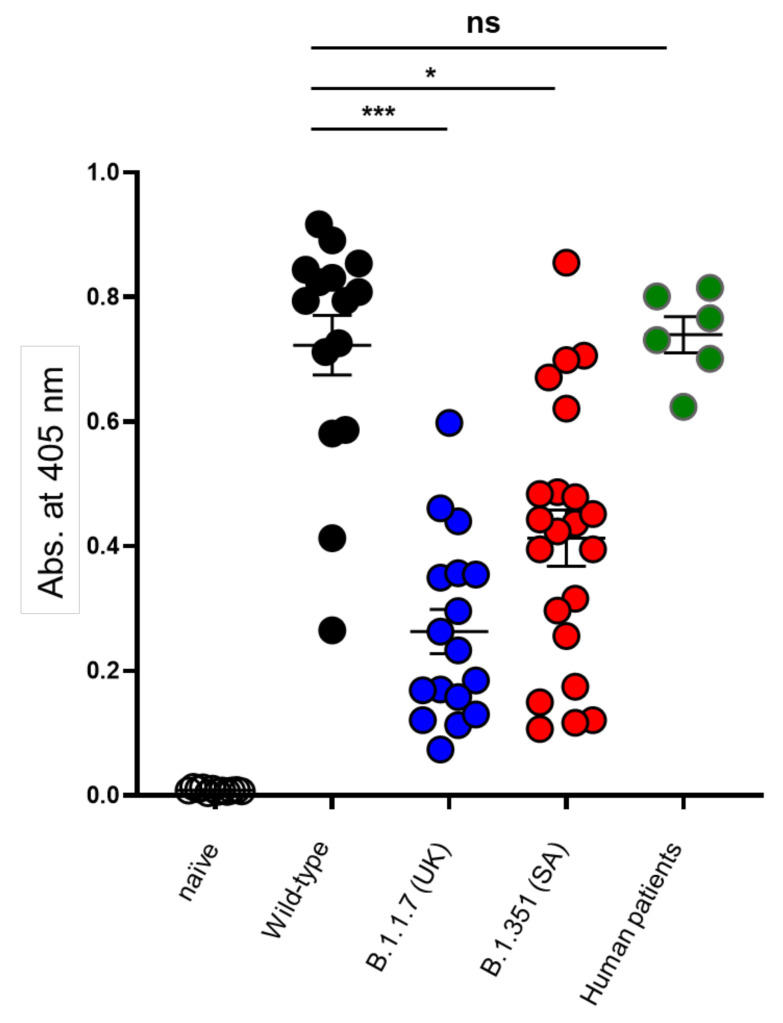
Humoral response against the spike glycoprotein among surviving mice and human patients. Endpoint values at 405 nm (−650 nm) representing the binding of naïve mouse sera (*n* = 12; open circles) and the sera obtained from mice surviving the infection with WT (*n* = 15; black circles), B.1.1.7 (*n* = 17; blue circles) and B.1.351 (*n* = 22; red circles) SARS-CoV-2 isolates towards the spike glycoprotein [28]. Human patients’ sera recovering from COVID-19 was also included (*n* = 6; green circles). Statistical differences were determined by a non-parametric Kruskal–Wallis with Dunn’s post hoc test. * *p* < 0.05, *** *p* < 0.005, ns = nonsignificant. The average background value based on 10 independent measurements was 0.0087 (range 0.004–0.014).

**Figure 2:**
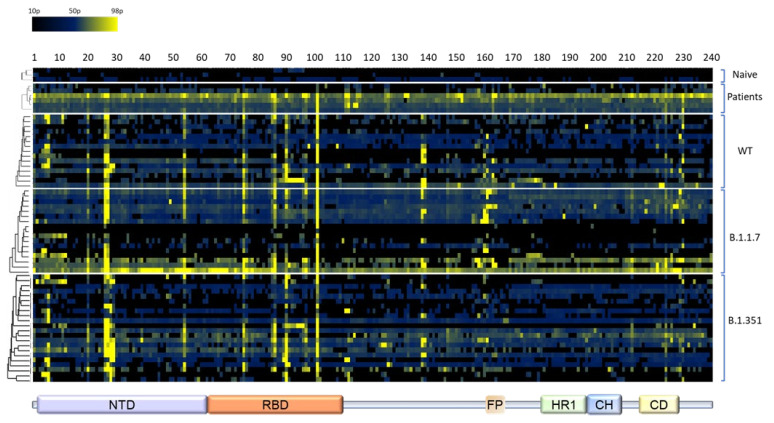
Heatmap plot of the binding results for the 240 peptides with various sera samples. Absorbance values at 405 nm marking the binding of convalescent serum antibodies to each one of the 240 peptides is presented by a heatmap. A graded 3-color scale of black (minimum—10th percentile), dark blue (midpoint—50th percentile) and yellow (maximum—98th percentile) was used. The peptide number is indicated on the top and the related spike domains depicted on the bottom. A hierarchal clustering using the average linkage method [29] was applied to the samples for each group in separate (top to bottom: naïve, COVID-19 patients, WT-infected mice, B.1.1.7-infected mice and B.1.351-infected mice).

Mouse sera samples were collected from K18-hACE2 transgenic (B6.Cg-Tg (K18-hACE2)2Prlmn/J HEMI; Jackson Laboratories, USA) mice (females, age 6–16 weeks) 21 days post-infection with WT/B.1.1.7/B.1.351 SARS-CoV-2 strains. These animals were mainly used in protection experiments that were previously reported [14,26,27]. While some of the samples were obtained from control (PBS-treated) animals that survived the infection (therefore without any therapeutic intervention promoting their survival), other samples were obtained from animals that survived owing to their previous treatment with neutralizing human antibodies, administered 48 h post-infection. The applicability of these samples for the current study, relayed on their positive mouse endogenous humoral response as well as the confirmed absence of the treated human Ab, as previously reported [26]. K18-hACE2 naïve mouse sera were used as a negative control. In summary, the screening included the following sera (see Figure 1 and Figure 2): (i) collected from 3 control naïve mice; (ii) 6 samples collected from infected human subjects; (iii) 15 samples from mice exposed to 200–500 PFU of the WT SARS-CoV-2 strain (9 PBS-treated and 6 antibody-treated mice [14,26,27]; (iv) 17 samples from mice exposed to 10 PFU of the B.1.1.7 SARS-CoV-2 strain (in this group only antibody–treated mice were included due to the high mortality [14]; and (v) 22 samples from mice exposed to the B.1.351 SARS-CoV-2 strain (a single PBS-treated and 9 antibody-treated mice exposed to 10^4^ PFU [14] and 3 PBS-treated and 9 antibody-treated mice exposed to 10^3^ PFU, reported here for the first time).

### 2.3. Enzyme-Linked Immunosorbent Assaay (ELISA)

Specific ELISA was performed against SARS-CoV-2 spike glycoprotein ectodomain (expressed and purified as described [28] for the evaluation of the endogenous humoral response of each tested serum sample. Maxisorp™ 96-well microtiter plates (Nunc, Roskilde, Denmark) were coated overnight with 1 µg/mL of spike protein in NaHCO3 buffer (50 mM, pH 9.6), washed, and blocked with PBT buffer (1xPBS, 0.05% Tween 20, 2% BSA). Samples were diluted 1:200 for determining their anti-spike titer (Figure 1). Human or mouse Abs were visualized by AP-conjugated donkey anti-human IgG (Jackson ImmunoResearch, Bar Harbor, ME, USA, Cat# 709-055-149, lot 130049; used at 1:1,000) or donkey anti-mouse IgG (H+L) minimal cross (Jackson ImmunoResearch, USA, Cat# 715-055-150, lot 142717; used at 1:2,000), respectively. PNPP substrate (Sigma, Rehovot, Israel, Cat# N1891) was applied for the reaction development. Washing steps were carried out with 1xPBST (1xPBS, 0.05% Tween 20) and all incubation were performed at room temperature.

### 2.4. Epitope Mapping

A total of 240 N-terminally biotinylated 15 amino-acid long peptides exhibiting 10 residue overlap with the adjacent peptides and covering the entire ectodomain of SARS-CoV-2 spike protein (1222 amino acids; Accession No. QHD43416) were purchased from JPT (Germany). Aliquots of peptides (1 mg/mL in DMSO) were diluted (10 µg/mL in 1xPBS) and added to streptavidin coated (2 µg/mL in 50 mM NaHCO3 buffer, pH 9.6) washed and blocked Maxisorp™ 96-well microtiter plates (3 plates per sample). All sera were diluted 1:300 in PBT and all incubations were performed at room temperature. Biotinylated spike protein (0.1 µg/mL in PBT) was added to the last raw of each test plate. As a standard, purified human anti-spike monoclonal antibody MD65 was added to the first well (10 µg/mL) of the two-fold serial dilution of the standard curve. All reactions were terminated when a 405 nm-absorbance value of 0.5 was reached in the well containing 0.31 µg/mL of the control MD65 antibody. This is imperative for correct assessment of the binding signal. Immune complexes were detected as indicated above.

### 2.5. Results Analysis

The absorbance values at 405 nm were plotted as a heatmap. The heatmap columns correspond to the 240 15-mer evaluated peptides, in consecutive order along the spike protein sequence. The heatmap rows represent the naïve, patients, WT, B.1.1.7 and B.1.351 samples. Hierarchical clustering was applied for each group, using the average linkage method [29].

### 2.6. Plaque Reduction Neutralization Test (PRNT)

Plaque reduction neutralization test (PRNT) was performed essentially as described in [28]. Vero E6 cells were seeded overnight at a density of 5 × 10^5^ cells/well in 12-well plates. Serum samples were 3-fold serially diluted in 400 μL MEM supplemented with 2% FBS, MEM non-essential amino acids, 2 mM L-glutamine, 100 Units/mL penicillin, 0.1 mg/mL streptomycin and 12.5 Units/mL Nystatin (Biological Industries, Israel). Then, 400 μL containing 300 PFU/mL of each SARS-CoV-2 strain, were then added to the serially diluted sera and the mixture incubated at 37 °C, 5% CO_2_ for 1 h. Next, 200 μL of each serum–virus mixture was added in duplicates to the seeded cells for 1 h. Virus mixture w/o serum was used as control. After this, 2 mL overlay (supplemented MEM containing 0.4% tragacanth (Sigma, Israel)) were then added to each well and plates were further incubated at 37 °C, 5% CO_2_ for 48 h for WT and B.1.351 strains or for 5 days for the B.1.1.7 strain. The number of plaques in each well was determined following media aspiration, cells fixation and staining with 1 mL of crystal violet (Biological Industries, Israel). Half-maximum inhibitory dilution (NT50) was defined as serum dilution at which the plaque number was reduced by 50%, compared to plaque number of the control (in the absence of serum).

## 3. Results

### 3.1. General Design of the Study and Antibodies Used for Epitope Mapping 

The objective of the study documented in this report was to determine whether antibodies generated in the context of the humoral immune response to individual SARS-CoV-2 variants differ with respect to their dominant targeted spike epitopes. Conceivably, differences in epitope recognition following infection with different variants may provide an explanation for the different susceptibility of the viral variants to vaccination and/or post-exposure immunotherapies, as well as for differences observed in the pathogenicity and contagiousness of the variants.

Accordingly, the linear epitope landscape of mouse humoral response against diverse variants of the SARS-CoV-2 was characterized using a comprehensive array for the epitope-mapping study. The array was probed with sera samples collected from K18-hACE2 transgenic mice surviving from an infection with either the BavPat1/2020SARS-CoV-2 isolate (Ref-SKU: 026V-03883; WT) and the two variants of concern (VOCs): B.1.1.7 Alpha and B.1.351 Beta. The mutated spike protein sequence of these VOCs is schematically depicted in Figure S1. Naïve K18-hACE2 sera were used as a negative control. Additionally, human sera, collected at the beginning of the COVID-19 pandemic from severe patients prior to the emergence of the VOCs, were used as a reference of the human infection. A total of 15, 17 and 22 serum samples, obtained from mice surviving the infection with either WT, B.1.1.7 or B.1.351 isolates, respectively, were inspected. Since the K18-hACE2 mice are considered to represent a stringent model of COVID-19 due to high mortality rates, analyzed sera were collected also from mice that were passively treated 48 h post infection [14]. Of note, none of these recombinant antibodies bound to any of the peptides in the epitope array (not shown). Furthermore, the sera employed was collected from the mice 21 days post-infection when the level of the therapeutic recombinant neutralizing antibodies in the sera was below the limit of detection, as corroborated by previous pharmacokinetic data [27]. In addition, the therapeutic antibodies were of human allotype and thus could not be recognized by the anti-mouse secondary antibodies used for probing the epitope array. The data in Figure 1, establishes that, the sera of surviving mice exhibited variability in binding to the spike glycoprotein. All sera exhibited significant anti-spike protein titers, sufficient for their implementation in the epitope mapping procedure. We noted that the humoral response against the WT strain was significantly higher than that observed following infection with either the B.1.1.7 or the B.1.351 variants, yet similar to the response quantified in human sera (Figure 1). Since the subsequent epitope mapping provides a qualitative rather than quantitative characterization of the response, and is not compromised by the sera titer, this issue was not further addressed.

### 3.2. Determination of the Epitope Landscape of the Spike Protein by Epitope Array Screening

**Figure 3:**
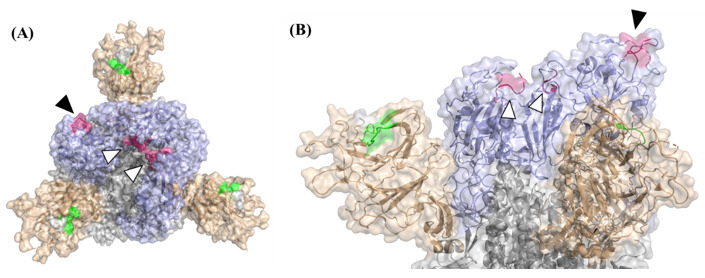
Spatial localization of the two most abundant cross-variant peptides on the spike trimer. Top (**A**) and side (**B**) Semi-transparent surface view of SARS-CoV-2 spike trimer (PBD 7C2l stripped off the 4A8 antibody). The N-terminal domain (NTD) is brown-colored and the receptor binding domain (RBD) is shown in purple. Other parts of the spike are colored in gray. The spatial localization of epitope 134QFCNDF139 is shown in green and epitope 501NGVGYQP507 is highlighted in dark pink. Black arrows mark the surface exposed epitope (when the RBD assumes the “up” position) and black-framed white arrows mark this epitope within the “closed” inaccessible position of the RBD. All analyses were performed by using the PyMol Molecular Graphics System (Version 1.7 Schrödinger, LLC (Portland, OR, USA)).

To ensure high coverage of linear epitopes in the spike protein ectodomain, the array used for analysis consisted of 240 individual 15-amino acid biotinylated peptides exhibiting 10 residues overlap. Sera collected from mice and human patients were diluted and applied on the peptide array (3 × 96 plates per sample). For standardization, pre-determined concentrations of purified human anti-spike antibody MD65 [27,28] were added to biotinylated recombinant spike-coated wells included in each test plate. Immune-complexes were detected by the appropriate anti-human or anti-mouse secondary antibodies conjugated to alkaline phosphatase and visualized by a standard p-Nitrophenyl phosphate assay. The absorbance values at 405 nm obtained in the assay were plotted as a heatmap (Figure 2) and a peptide was defined as a “positive” binder when the signal intensity was 4 times that of the averaged background values exhibited by wells including entire spike protein without any primary antibody. The complete peptide list included in this study and the frequency of the binding sera among the various experimental groups is depicted in Table S1. The percentage of sera binding a particular epitope served as a quantitative reflection of its prevalence in the context of the response to infection and immunodominance of epitopes was considered for peptides that were found ”positive” in at least 20% of the mouse sera. The analysis enabled the identification of several immune-dominant epitopes (summarized in Table 1) at positions mapped mainly to the NTD and RBD, regardless of the SARS-CoV-2 variant used to infect the mice. Notably, peptides 26 (126VVIKVCEFQFCNDPF140) and 27 (131CEFQFCNDPFLGVYY145), covering amino acids 126–140 (NTD), were detected in 33–76% of surviving mice. Close inspection of the solved structure of the SARS-CoV-2 spike glycoprotein (7c2l; [10]) revealed that only 6 amino acids shared by these two peptides (134QFCNDF139) are exposed on the surface of the spike glycoprotein (Figure 3). Interestingly, the two successive peptides 28 (141CNDPFLGVYYHKNNK155) and 29 (146LGVYYHKNNKSWMES160) were exclusively detected by mice recovering from infection with the B.1.351variant (Table S1). This observation is compatible with previous studies that have shown that the NTD in the B.1.351 variant spike glycoprotein undergoes conformational modifications that probably expose “buried” sequences of the spike glycoprotein to the immune system on one hand, and severely affect the neutralization capabilities of anti-NTD mAbs, on the other hand [2]. Remarkably, peptide 101 (501NGVGYQPYRVVVLSF515) appears to represent a highly immunodominant epitope in the RBD, as it was recognized by antibodies from 55–88% recovering mice and 67% of the patients (Figure 2 and Table 1). 

This peptide includes residue N501 that directly interacts with the hACE2. The mutational conversion of this residue to tyrosine (N501Y) is encountered in the two VOCs tested in this study, suggesting a possible contribution to the high transmissibility of these variants [30]. Model analysis which enabled superimposition of this peptide on the solved structure of the SARS-CoV-2 spike glycoprotein (7c2l), established that the first seven amino acids (501NGVGYQP507) are exposed on the surface when the RBD adopts the “Up” position (Figure 3). Interestingly, the preceding peptide in the array (peptide #102; 496GFQPTNGVGYQPYRV510) which includes the 501NGVGYQP507 amino acids, was not bound by any of the examined sera (Table S1). Therefore, we suggest that in spite of the fact that the survey is directed to the distinction of linear epitopes, the peptide 501NGVGYQPYRVVVLSF515 folded in the array allows the recognition of the 7 residues peptide 501NGVGYQP507 by the mouse and human sera. A similar folding probably did not occur in the preceding overlapping peptide. Of note, none of the peptides was detected by naïve mouse sera, supporting the notion that the assay evidenced bona-fide epitopes whose immune recognition is elicited in the context of the response to SARS-CoV-2 infection (Figure 2).

Close examination of the peptides that were found as immune-dominant cross-variant binders revealed that some of them include positions that are mutated in the VOCs examined in this study. Peptide 27 harbors tyrosine 144 (Y144) that is deleted in the B.1.1.7 variant while peptide 101 includes asparagine 501 (N501) that is mutated to tyrosine (N501Y) in both B.1.1.7 and B.1.351 variants (Table 1). Yet, the ability of sera obtained from the three mouse experimental groups to bind peptides 27 and 101 was well maintained, suggesting that these mutations influence at a low extent the spike protein linear epitope landscape (Table 1). To examine this aspect for the mutated peptides, we examined in parallel the binding of individual mouse sera to the “WT” peptides and to the respective peptides that harbor the Δ144 deletion and the N501Y mutation (Table 2). As hypothesized, among the groups infected with the three variants similar proportions of sera were found as positive binders of the N501Y mutated peptide and the comparable “WT” peptide (Table 2). Unexpectedly, the peptides that included the Δ144 mutation were detected at much lower frequency than the comparable “WT” peptides, even among mice that recovered from infection with the B.1.1.7 variant (Table 2). The reason for this result is not clear yet it is conceivable that the inclusion of histidine in the peptide had a detrimental effect on the folding /net charge of the peptide that resulted in the loss of binding of the convalescent sera.

### 3.3. Cross-Variant Neutralization by Plaque-Reduction Neutralization Test

**Figure 4:**
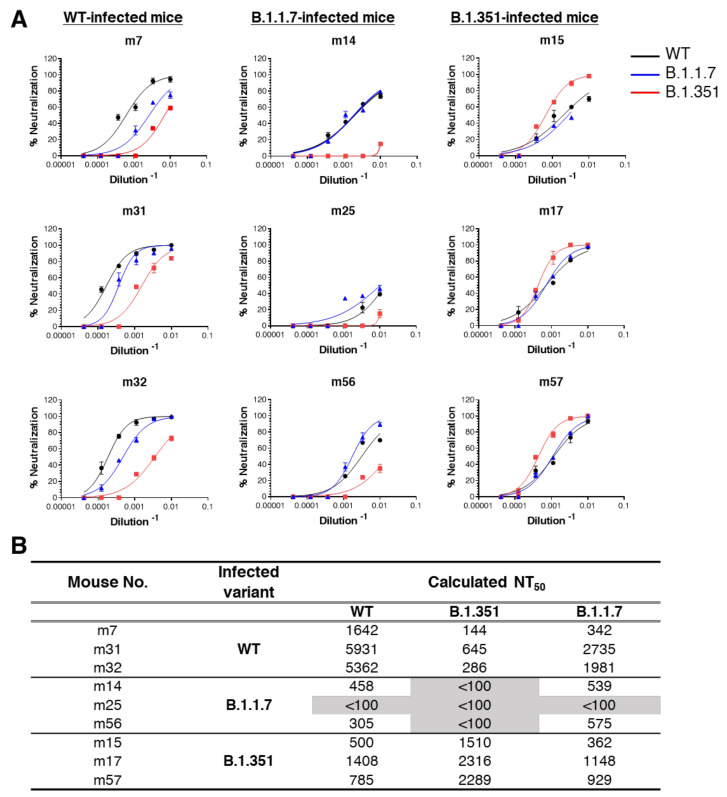
Cross-neutralization of SARS-CoV-2 WT, B.1.1.7 and B.1.351 variants. The in vitro neutralization capacity of serum samples collected from WT, B.1.1.7 or B.1.351 SARS-CoV-2 infected mice, was assessed by plaque reduction neutralization test (PRNT). The neutralization ability of each sample (at indicated dilutions) was assessed in duplicates against SARS-CoV-2 WT (black), B.1.1.7 (blue) and B.1.351 (red) variant. (**A**) Results are expressed as percent inhibition compared to control without serum. (**B**) Summary of the calculated NT50 values (dilution-1). NT50 <100 indicates low neutralization capacity, emphasized by gray shading.

The overall similar linear epitope landscape generated by mice surviving the infection with WT SARS-CoV-2 isolate as well as the B.1.1.7 and the B.1.351 variants suggested that the polyclonal immune response generated in the mice may compensate for the differences in the structures of the spike glycoprotein that is evident between the three virus isolates [31]. To address this issue, a cross-neutralization efficacy assay by the plaque reduction neutralization test (PRNT) was performed. Nine serum samples collected from mice infected with either WT SARS-CoV-2 or the two VOCs: B.1.1.7 and B.1.351 strains were examined. VeroE6 cells were infected individually with each of the three SARS-CoV-2 strains and the neutralizing potency of each serum sample was calculated as the dilution that reduced plaque formation to 50% (NT50) for each variant. Figure 4, shows the titer-dependent neutralization profile (panel A) as well as the calculated NT50 values (panel B) of the nine representative serum samples that generated the highest anti-spike titers. As anticipated, all sera samples were most effective in neutralizing the virus variant to which the respective animals were exposed, compared to the other strains. Sera derived from WT-infected mice exhibited 2- to 5-fold and 9- to 19-fold decrease in NT50 values when tested against B.1.1.7 or B.1.351, respectively. In line with this result, while a minimal reduction (less than 2-fold) in the neutralizing potency of sera obtained from B.1.1.7-infected mice towards the WT strain was observed, the neutralization potency of these was 5-fold lower when tested against the B.1.351 variant. Finally, the neutralization potency of sera collected from B.1.351-infected mice, showed 2- to 4-fold reduction in neutralization against both WT and the B.1.1.7 variant (Figure 4B).

## 4. Discussion

The ongoing emergence of SARS-CoV-2 VOCs and the possibility that some of these variants exhibit inferior susceptibility to the recently FDA-licensed and already widely used COVID-19 vaccines, have a tremendous impact on the progress of the current pandemic and consequently constitute one of the major drawbacks in globally controlling the disease. The current study addresses the variability in the immune responses elicited by various mutants as a pre-requisite for the development of universally efficient countermeasures. One of the methods to characterize the multi-epitope characteristic of the immune response elicited by infection (or vaccination) is to generate a “map” detailing the antibody specificity towards an array of overlapping peptides that fully cover the spike glycoprotein. Indeed, several studies undertook this approach to characterize the epitope “landscape” that developed in sick or convalescent COVID-19 patients [9,18,32,33,34,35]. Other reports documented bioinformatic strategies to identify potential immunodominant epitopes that may influence the efficacy of COVID-19 immunity [20,21,23]. Most of these studies were conducted by employing serum samples collected from individual infected with the primordial WT viral isolate [11,18,25,32]. In the present study, the K18-hACE2 mouse model of COVID-19 was implemented to compare the humoral response elicited following exposure to the WT isolate as well as to the B.1.1.7 and B.1.351 VOCs. K18-hACE2 transgenic mice, engineered to express the human version of the ACE2 receptor were previously shown to represent a valuable COVID-19 model, faithfully recapitulating the viral virulence and accordingly, broadly used for SARS-CoV-2 pathogenesis studies and anti-viral therapy efficacy assessment [27,36,37,38,39,40]. 

The study enabled identification of several immunodominant (ID) positions along the spike glycoprotein that were recognized by sera collected from infected mice regardless of the variant used for infection (Figure 2, Table 1). The majority of these cross-variant ID positions were assigned to the S1 portion of the spike glycoprotein, strongly supporting its importance in the mounting of protective humoral response against COVID-19. Interestingly, the two most abundant cross-variant ID positions that reside in the NTD (amino acids 126–145) and the RBD (amino acid 501–515) were not detected by previous experimental studies that also examined the specificity of the humoral response against an array of peptides derived from the spike glycoprotein [18,32,33]. The reason for this discrepancy may stem from the overall different characteristics of the experimental systems such as the length of the peptides, the degree of overlap between adjacent peptides, the sensitivity of the detection protocol and also the present use of inbred mice as a source of sera for the immune response evaluation. On the other hand, peptides that emerged as epitopes at less than 20% of all animal groups, such as peptide #5 21RTQLPPAYTNSFTRG35, peptide #6 26PAYTNSFTRGVYYPD40 and peptide #90 456GGNYNYLYRLFRKSN460 (see Table S1) were also evidenced by previous studies, [9,32,33]. Furthermore, four out of the nine ID sites identified in the study of B.Z. Wang et al. 2020 were found to contain linear epitopes that overlap in part with the cross-variant sequences described in the current study, although not all of them recognized at 20% of the mice (see peptides #5, #6, #75, #90 and #91; Table S1). The data in the current study are also supported by several computational-based studies that employed bioinformatic tools to predict the localization of possible linear epitopes along the SARS-CoV-2 spike ectodomain [20,24]. A recent comprehensive study predicted the existence of nine epitopes each composed of a single or several linear sequences [20]. Six out of these nine epitopes overlap in part with the linear epitopes that we have found, including the two most abundant cross-variants that span amino acids 126–145 and 501–515 (Table 1). Another study characterized the binding sites of four monoclonal antibodies obtained from COVID-19 patients [24]. All of these four epitopes share linear sequences with the cross-variant peptides that are described in the current manuscript. This overlap between linear epitopes that were predicted by computational-based and structural-based studies to those found by the experimental approach, substantiates the validity of the findings reported here. 

It is notable that infection with the three SARS-CoV-2 variants yielded a similar pattern of ID peptides considering that peptides 131CEFQFCNDPFLGVYY145 and 501NGVGYQPYRVVVLSF515, both of which being frequently recognized by all variants, include the residues Y144 and N501 (respectively) that are modified in some of the VOCs. In studies involving convalescent human sera, these mutations, together with additional genetic changes, were suggested to be associated with higher transmissibility and superior immune escape ability [2,30,41,42]. The observation that the N501Y mutation did not alter the frequency of epitope-recognition by antibodies collected from mutant-infected mice further supports the conclusion that the three SARS-CoV-2 isolates elicited a similar humoral response (Figure 2). Conversely, the loss of binding ability of most of the analyzed sera against the peptide that harbors the ΔY144 mutation (131CEFQFCNDPFLGVYH146), suggests that additional residues participate in efficient recognition of this mutated epitope by the various sera. We hypothesize that the histidine residue accompanying the deletion of Y144 may have a detrimental effect on the binding efficacy of all sera, even those obtained from the animals that were infected with the B.1.1.7 variant (Table 2).

## 5. Conclusions

The data establishing an overall similar epitope landscape elicited by exposure to the three isolates suggested that protection conferred upon vaccination with the WT strain is not compromised by infection with other variants. Indeed, we bring evidence that the various sera cross-neutralize the WT and VOCs under study (Figure 3). Our results are in line with those reported by Tang J. et al. 2021 following a study addressing the neutralization potential of several hyper-immunoglobulin and convalescent plasma preparations generated against WT SARS-CoV-2 towards the B.1.1.7 and B.1.351 VOCs [43]. Furthermore, the individual ability of monoclonal antibodies generated against the WT spike glycoprotein to cross-neutralize (in vitro) and cross-protect (in vivo) against the B.1.1.7 and B.1.351 VOCs was recently reported [14].

Taken together, these studies addressing the universality and robustness of the immune-prophylactics and therapies based on the WT strain are valuable for the development of countermeasures to the current pandemic.

## Figures and Tables

**Table 1:** Fraction of “positive” linear peptides detected by sera obtained from infected mice with the three SARS-CoV-2 isolates and among human patients.

Peptide No.	Spike aa Position	Spike Domain	Peptide aa Sequence	Fraction of Positive *			
				WT (*n* = 15)	B.1.1.7 (*n* = 17)	B.1.351 (*n* = 22)	Patients (*n* = 6)
26	126–140	NTD	VVIKVCEFQFCNDPF	7/15	13/17	10/22	1/6
27	131–145	NTD	CEFQFCNDPFLGVYY	5/15	8/17	11/22	1/6
75	371–385	RBD	SASFSTFKCYGVSPT	4/15	7/17	5/22	1/6
86	426–440	RBD	PDDFTGCVIAWNSNN	3/15	6/17	5/22	2/6
101	501–515	RBD	NGVGYQPYRVVVLSF	13/15	15/17	11/22	4/6
138	686–700	S2	SVASQSIIAYTMSLG	5/15	7/17	5/22	0/6

* Positive peptide recognition of serum sample, considered when the signal intensity was 4 times that of the background. Fraction of positive mice were determined individually for each experimental group.

**Table 2:** Fraction of “positive” linear WT and the mutated peptides detected by sera obtained from convalescent mice obtained after infection with the three SARS-CoV-2 isolates.

Peptide Type	Spike aa Position	Peptide aa Sequence *	WT	B.1.1.7	B.1.351
	131–145	CEFQFCNDPFLGVY-H	1/15	1/17	4/22
Δ144	136–150	CNDPFLGVY-HKNNKS	0	0	2/22
	141–155	LGVY-HKNNKSWMESE	0	0	2/22
	131–145	CEFQFCNDPFLGVYY	10/15	13/17	10/22
WT	136–150	CNDPFLGVYYHKNNK	0	0	13/22
	141–155	LGVYYHKNNKSWMES	0	0	7/22
	491–505	PLQSYGFQPTYGVGY	0	0	2/22
N501Y	496–510	GFQPTYGVGYQPYRV	0	0	2/22
	501–515	YGVGYQPYRVVVLSF	10/15	15/17	10/22
	491–505	PLQSYGFQPTNGVGY	0	0	2/22
WT	496–510	GFQPTNGVGYQPYRV	0	0	2/22
	501–515	NGVGYQPYRVVVLSF	8/15	13/17	8/22

* All peptides were maintained at a length of 15 amino acids. Tyrosine 501 is shown in bold. Positive peptides were determined as indicated in text. In the case of the Δ 144 mutant, the position of the deletion is indicated by a middle-hyphen.

## Data Availability

Raw data pertaining to the study may be obtained upon request.

## Supplementary Materials

The following supporting information can be downloaded at: https://www.mdpi.com/article/10.3390/vaccines10020251/s1, Figure S1: Schematic illustration of the spike protein of the B.1.1.7 and B.1.351 VOCs. Table S1: Peptides included in this study and the percentage of positive sera among the experimental groups.
