# Supplementary material for: Immunodominant Linear B-Cell Epitopes of SARS-CoV-2 Spike, Identified by Sera from K18-hACE2 Mice Infected with the WT or Variant Viruses
Source: Vaccines (Basel). 2022 Feb 7;10(2):251. doi: 10.3390/vaccines10020251 (PMC8875268; doi:10.3390/vaccines10020251)

Figure S1: Schematic illustration of the spike protein of the B.1.1.7 and B.1.351 VOCs. SARS-CoV-2 spike protein ectodomain consists of a signal peptide (SP), S1 and S2 subunits. The S1 subunit is composed of the N-terminal domain (NTD) and the receptor binding domain (RBD). The S2 subunit composed of a fusion peptide (FP), heptad repeats 1 and 2 (HR1 and HR2), central helix (CH) and connector domain (CD). Mutated and deleted amino acids between the WT and the two variant spike proteins are shown above.

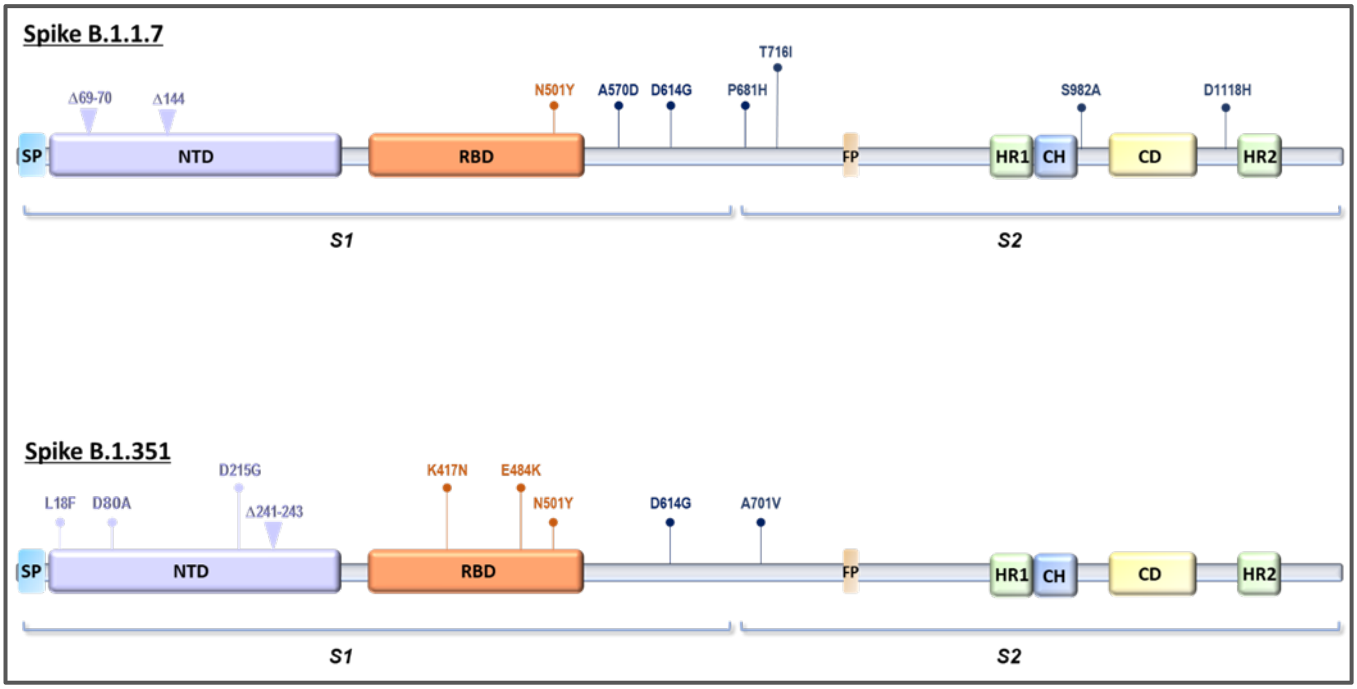

Supplement: Supplementary file 1 [file vaccines-10-00251-s001.zip › Figure S1.pdf]
