# Supplementary material for: Immunodominant Linear B-Cell Epitopes of SARS-CoV-2 Spike, Identified by Sera from K18-hACE2 Mice Infected with the WT or Variant Viruses
Source: Vaccines (Basel). 2022 Feb 7;10(2):251. doi: 10.3390/vaccines10020251 (PMC8875268; doi:10.3390/vaccines10020251)
Supplement: Supplementary file 1 [file vaccines-10-00251-s001.zip › Table S1.pdf]

Table S1. Peptides included in this study and the percentage of positive sera among the experimental groups.

Peptide No.	Spike aa start-position	Peptide sequence	Spike aa end-position	Number of "positive" sera samples		
				WT (n=15)	B.1.1.7 (n=17)	B.1.351 (n=22)
1	1	MFVFLVLLPLVSSQC	15	0	2	0
2	6	VLLPLVSSQCVNLTT	20	0	0	1
3	11	VSSQCVNLTTRTQLP	25	0	0	0
4	16	VNLTTRTQLPPAYTN	30	0	1	1
5	21	RTQLPPAYTNSFTRG	35	3	1	3
6	26	PAYTNSFTRGVYYPD	40	5	0	10
7	31	SFTRGVYYPDKVFRS	45	0	0	0
8	36	VYYPDKVFRSSVLHS	50	0	0	0
9	41	KVFRSSVLHSTQDLF	55	0	0	0
10	46	SVLHSTQDLFLPFFS	60	0	1	0
11	51	TQDLFLPFFSNVTWF	65	1	1	1
12	56	LPFFSNVTWFHAIHV	70	0	2	1
13	61	NVTWFHAIHVSGTNG	75	0	0	0
14	66	HAIHVSGTNGTKRFD	80	0	0	0
15	71	SGTNGTKRFDNPVLP	85	0	0	0
16	76	TKRFDNPVLPFNDGV	90	0	0	0
17	81	NPVLPFNDGVYFAST	95	0	0	0
18	86	FNDGVYFASTEKSNI	100	0	0	0
19	91	YFASTEKSNIIRGWI	105	0	0	0
20	96	EKSNIIRGWIFGTTL	110	1	2	1
21	101	IRGWIFGTTLDSKTQ	115	0	0	0
22	106	FGTTLDSKTQSLLIV	120	0	0	0
23	111	DSKTQSLLIVNNATN	125	0	0	0
24	116	SLLIVNNATNVVIKV	130	0	0	0
25	121	NNATNVVIKVCEFQF	135	0	0	0
26	126	VVIKVCEFQFCNDPF	140	7	13	10
27	131	CEFQFCNDPFLGVYY	145	5	8	11
28	136	CNDPFLGVYYHKNNK	150	2	0	11
29	141	LGVYYHKNNKSWMES	155	1	0	6
30	146	HKNNKSWMESEFRVY	160	0	0	0
31	151	SWMESEFRVYSSANN	165	0	1	0
32	156	EFRVYSSANNCTFEY	170	0	1	0
33	161	SSANNCTFEYVSQPF	175	0	1	0
34	166	CTFEYVSQPFLMDLE	180	0	1	0
35	171	VSQPFLMDLEGKQGN	185	0	0	0
36	176	LMDLEGKQGNFKNLR	190	0	0	0
37	181	GKQGNFKNLREFVFK	195	0	0	0
38	186	FKNLREFVFKNIDGY	200	0	0	0
39	191	EFVFKNIDGYFKIYS	205	0	1	1
40	196	NIDGYFKIYSKHTPI	210	0	1	0
41	201	FKIYSKHTPINLVRD	215	0	1	0
42	206	KHTPINLVRDLPQGF	220	0	1	0
43	211	NLVRDLPQGFSALEP	225	0	1	0
44	216	LPQGFSALEPLVDLP	230	0	1	0
45	221	SALEPLVDLPIGINI	235	0	1	0
46	226	LVDLPIGINITRFQT	240	0	1	0
47	231	IGINITRFQTLLALH	245	0	0	0
48	236	TRFQTLLALHRSYLT	250	0	0	0
49	241	LLALHRSYLTPGDSS	255	0	1	0

50	246	RSYLTPGDSSSGWTA	260	0	0	0
51	251	PGDSSSGWTAGAAAY	265	0	1	0
52	256	SGWTAGAAAYYVGYL	270	0	1	0
53	261	GAAAYYVGYLQPRTF	275	0	1	0
54	266	YVGYLQPRTFLLKYN	280	2	5	4
55	271	QPRTFLLKYNENGTI	285	0	1	0
56	276	LLKYNENGTITDAVD	290	0	1	0
57	281	ENGTITDAVDCALDP	295	0	1	0
58	286	TDAVDCALDPLSETK	300	0	0	0
59	291	CALDPLSETKCTLKS	305	0	0	0
60	296	LSETKCTLKSFTVEK	310	0	0	0
61	301	CTLKSFTVEKGIYQT	315	0	0	0
62	306	FTVEKGIYQTSNFRV	320	0	0	0
63	311	GIYQTSNFRVQPTES	325	0	1	0
64	316	SNFRVQPTESIVRFP	330	0	1	0
65	321	QPTESIVRFPNITNL	335	0	1	0
66	326	IVRFPNITNLCPFGE	340	0	1	0
67	331	NITNLCPFGEVFNAT	345	0	1	0
68	336	CPFGEVFNATRFASV	350	0	1	0
69	341	VFNATRFASVYAWNR	355	0	1	0
70	346	RFASVYAWNRKRISN	360	0	0	0
71	351	YAWNRKRISNCVADY	365	0	0	0
72	356	KRISNCVADYSVLYN	370	0	1	0
73	361	CVADYSVLYNSASFS	375	0	0	0
74	366	SVLYNSASFSTFKCY	380	0	0	0
75	371	SASFSTFKCYGVSPT	385	4	7	5
76	376	TFKCYGVSPTKLNDL	390	0	2	0
77	381	GVSPTKLNDLCFTNV	395	0	1	0
78	386	KLNDLCFTNVYADSF	400	0	1	0
79	391	CFTNVYADSFVIRGD	405	0	0	0
80	396	YADSFVIRGDEVRQI	410	0	0	0
81	401	VIRGDEVRQIAPGQT	415	0	0	0
82	406	EVRQIAPGQTGKIAD	420	0	0	0
83	411	APGQTGKIADYNYKL	425	0	0	0
84	416	GKIADYNYKLPDDFT	430	0	0	0
85	421	YNYKLPDDFTGCVIA	435	0	1	0
86	426	PDDFTGCVIAWNSNN	440	3	6	5
87	431	GCVIAWNSNNLDSKV	445	0	0	0
88	436	WNSNNLDSKVGGNYN	450	0	0	0
89	441	LDSKVGGNYNYLYRL	455	0	0	1
90	446	GGNYNYLYRLFRKSN	460	6	2	13
91	451	YLYRLFRKSNLKPFE	465	1	1	2
92	456	FRKSNLKPFERDIST	470	1	0	0
93	461	LKPFERDISTEIYQA	475	1	0	0
94	466	RDISTEIYQAGSTPC	480	1	0	1
95	471	EIYQAGSTPCNGVEG	485	1	0	1
96	476	GSTPCNGVEGFNCYF	490	1	1	1
97	481	NGVEGFNCYFPLQSY	495	0	3	1
98	486	FNCYFPLQSYGFQPT	500	1	0	0
99	491	PLQSYGFQPTNGVGY	505	0	0	0
100	496	GFQPTNGVGYQPYRV	510	0	0	0
101	501	NGVGYQPYRVVVLSF	515	13	15	12
102	506	QPYRVVVLSFELLHA	520	0	0	0
103	511	VVLSFELLHAPATVC	525	0	0	0
104	516	ELLHAPATVCGPKKS	530	0	0	0
105	521	PATVCGPKKSTNLVK	535	0	0	0

106	526	GPKKSTNLVKNKCVN	540	0	0	0
107	531	TNLVKNKCVNFNFNG	545	0	0	0
108	536	NKCVNFNFNGLTGTG	550	1	0	0
109	541	FNFNGLTGTGVLTES	555	0	0	0
110	546	LTGTGVLTESNKKFL	560	0	0	0
111	551	VLTESNKKFLPFQQF	565	0	0	2
112	556	NKKFLPFQQFGRDIA	570	0	0	2
113	561	PFQQFGRDIADTTDA	575	0	0	0
114	566	GRDIADTTDAVRDPQ	580	0	0	1
115	571	DTTDAVRDPQTLEIL	585	0	0	0
116	576	VRDPQTLEILDITPC	590	0	0	0
117	581	TLEILDITPCSFGGV	595	0	0	0
118	586	DITPCSFGGVSVITP	600	0	0	0
119	591	SFGGVSVITPGTNTS	605	0	0	0
120	596	SVITPGTNTSNQVAV	610	0	0	0
121	601	GTNTSNQVAVLYQDV	615	0	0	0
122	606	NQVAVLYQDVNCTEV	620	0	0	0
123	611	LYQDVNCTEVPVAIH	625	0	0	0
124	616	NCTEVPVAIHADQLT	630	0	0	1
125	621	PVAIHADQLTPTWRV	635	0	0	1
126	626	ADQLTPTWRVYSTGS	640	0	0	1
127	631	PTWRVYSTGSNVFQT	645	0	0	0
128	636	YSTGSNVFQTRAGCL	650	0	0	0
129	641	NVFQTRAGCLIGAEH	655	0	0	0
130	646	RAGCLIGAEHVNNSY	660	0	0	0
131	651	IGAEHVNNSYECDIP	665	0	0	0
132	656	VNNSYECDIPIGAGI	670	0	0	0
133	661	ECDIPIGAGICASYQ	675	0	0	0
134	666	IGAGICASYQTQTNS	680	0	0	0
135	671	CASYQTQTNSPRRAR	685	0	0	0
136	676	TQTNSPRRARSVASQ	690	0	0	0
137	681	PRRARSVASQSIIAY	695	0	0	0
138	686	SVASQSIIAYTMSLG	700	5	7	5
139	691	SIIAYTMSLGAENSV	705	4	2	2
140	696	TMSLGAENSVAYSNN	710	0	0	0
141	701	AENSVAYSNNSIAIP	715	0	0	0
142	706	AYSNNSIAIPTNFTI	720	0	0	0
143	711	SIAIPTNFTISVTTE	725	0	0	0
144	716	TNFTISVTTEILPVS	730	0	0	0
145	721	SVTTEILPVSMTKTS	735	0	0	0
146	726	ILPVSMTKTSVDCTM	740	0	0	0
147	731	MTKTSVDCTMYICGD	745	0	1	0
148	736	VDCTMYICGDSTECS	750	0	0	0
149	741	YICGDSTECSNLLLQ	755	0	0	0
150	746	STECSNLLLQYGSFC	760	0	0	0
151	751	NLLLQYGSFCTQLNR	765	0	0	0
152	756	YGSFCTQLNRALTGI	770	0	0	0
153	761	TQLNRALTGIAVEQD	775	0	0	0
154	766	ALTGIAVEQDKNTQE	780	0	0	0
155	771	AVEQDKNTQEVFAQV	785	0	0	0
156	776	KNTQEVFAQVKQIYK	790	0	0	0
157	781	VFAQVKQIYKTPPIK	795	0	1	0
158	786	KQIYKTPPIKDFGGF	800	0	2	0
159	791	TPPIKDFGGFNFSQI	805	0	1	0
160	796	DFGGFNFSQILPDPS	810	1	4	0
161	801	NFSQILPDPSKPSKR	815	2	6	1

162	806	LPDPSKPSKRSFIED	820	0	2	0
163	811	KPSKRSFIEDLLFNK	825	2	2	0
164	816	SFIEDLLFNKVTLAD	830	0	2	0
165	821	LLFNKVTLADAGFIK	835	0	0	0
166	826	VTLADAGFIKQYGDC	840	0	0	0
167	831	AGFIKQYGDCLGDIA	845	0	0	0
168	836	QYGDCLGDIAARDLI	850	0	0	0
169	841	LGDIAARDLICAQKF	855	0	0	0
170	846	ARDLICAQKFNGLTV	860	0	0	0
171	851	CAQKFNGLTVLPPLL	865	0	0	0
172	856	NGLTVLPPLLTDEMI	870	0	0	0
173	861	LPPLLTDEMIAQYTS	875	0	0	0
174	866	TDEMIAQYTSALLAG	880	0	0	0
175	871	AQYTSALLAGTITSG	885	0	0	0
176	876	ALLAGTITSGWTFGA	890	0	0	0
177	881	TITSGWTFGAGAALQ	895	0	0	0
178	886	WTFGAGAALQIPFAM	900	1	0	0
179	891	GAALQIPFAMQMAYR	905	0	0	0
180	896	IPFAMQMAYRFNGIG	910	0	0	0
181	901	QMAYRFNGIGVTQNV	915	0	0	0
182	906	FNGIGVTQNVLYENQ	920	0	0	0
183	911	VTQNVLYENQKLIAN	925	0	0	0
184	916	LYENQKLIANQFNSA	930	0	0	0
185	921	KLIANQFNSAIGKIQ	935	1	0	0
186	926	QFNSAIGKIQDSLSS	940	0	0	0
187	931	IGKIQDSLSSTASAL	945	0	0	0
188	936	DSLSSTASALGKLQD	950	0	1	0
189	941	TASALGKLQDVVNQN	955	0	0	0
190	946	GKLQDVVNQNAQALN	960	0	0	0
191	951	VVNQNAQALNTLVKQ	965	0	0	0
192	956	AQALNTLVKQLSSNF	970	0	0	0
193	961	TLVKQLSSNFGAISS	975	0	0	0
194	966	LSSNFGAISSVLNDI	980	0	0	0
195	971	GAISSVLNDILSRLD	985	0	0	0
196	976	VLNDILSRLDKVEAE	990	1	0	0
197	981	LSRLDKVEAEVQIDR	995	0	0	0
198	986	KVEAEVQIDRLITGR	1000	0	0	0
199	991	VQIDRLITGRLQSLQ	1005	0	0	0
200	996	LITGRLQSLQTYVTQ	1010	0	0	0
201	1001	LQSLQTYVTQQLIRA	1015	0	0	0
202	1006	TYVTQQLIRAAEIRA	1020	0	0	0
203	1011	QLIRAAEIRASANLA	1025	0	0	0
204	1016	AEIRASANLAATKMS	1030	0	0	0
205	1021	SANLAATKMSECVLG	1035	0	0	0
206	1026	ATKMSECVLGQSKRV	1040	0	0	0
207	1031	ECVLGQSKRVDFCGK	1045	0	0	0
208	1036	QSKRVDFCGKGYHLM	1050	0	0	0
209	1041	DFCGKGYHLMSFPQS	1055	0	0	0
210	1046	GYHLMSFPQSAPHGV	1060	0	0	0
211	1051	SFPQSAPHGVVFLHV	1065	0	0	0
212	1056	APHGVVFLHVTYVPA	1070	0	2	0
213	1061	VFLHVTYVPAQEKNF	1075	0	0	0
214	1066	TYVPAQEKNFTTAPA	1080	0	0	0
215	1071	QEKNFTTAPAICHDG	1085	0	0	0
216	1076	TTAPAICHDGKAHFP	1090	0	0	0
217	1081	ICHDGKAHFPREGVF	1095	0	1	0

218	1086	KAHFPREGVFVSNGT	1100	0	0	0
219	1091	REGVFVSNGTHWFVT	1105	0	0	0
220	1096	VSNGTHWFVTQRNFY	1110	0	0	0
221	1101	HWFVTQRNFYEPQII	1115	0	2	0
222	1106	QRNFYEPQIITTDNT	1120	1	0	0
223	1111	EPQIITTDNTFVSGN	1125	1	0	0
224	1116	TTDNTFVSGNCDVVI	1130	0	3	0
225	1121	FVSGNCDVVIGIVNN	1135	0	0	0
226	1126	CDVVIGIVNNTVYDP	1140	0	2	1
227	1131	GIVNNTVYDPLQPEL	1145	0	2	0
228	1136	TVYDPLQPELDSFKE	1150	0	2	0
229	1141	LQPELDSFKEELDKY	1155	1	3	0
230	1146	DSFKEELDKYFKNHT	1160	2	2	1
231	1151	ELDKYFKNHTSPDVD	1165	0	0	0
232	1156	FKNHTSPDVDLGDIS	1170	0	0	0
233	1161	SPDVDLGDISGINAS	1175	0	0	0
234	1166	LGDISGINASVVNIQ	1180	0	0	1
235	1171	GINASVVNIQKEIDR	1185	0	0	0
236	1176	VVNIQKEIDRLNEVA	1190	0	0	0
237	1181	KEIDRLNEVAKNLNE	1195	0	0	0
238	1186	LNEVAKNLNESLIDL	1200	0	0	0
239	1191	KNLNESLIDLQELGK	1205	0	0	0
240	1196	LNESLIDLQELGKYE	1210	0	0	0
